# Effect of sphincter of Oddi dysfunction on the abundance of biliary microbiota (biliary microecology) in patients with common bile duct stones

**DOI:** 10.3389/fcimb.2022.1001441

**Published:** 2022-12-07

**Authors:** Linxun Liu, Zhanxue Zhao, Xiaofan Hou, Jindu Wu

**Affiliations:** ^1^ Department of General Surgery, Qinghai Provincial People’s Hospital, Xining, Qinghai, China; ^2^ Graduate College of Qinghai University, Xining, Qinghai, China

**Keywords:** choledocholithiasis, sphincter of Oddi, microbiota, bile duct stones, biliary microecology

## Abstract

**Objective:**

Biliary calculi, a common benign disease of the gastrointestinal tract, are affected by multiple factors, including diet, lifestyle, living environment, and personal and genetic background. Its occurrence is believed to be related to a change in biliary microbiota. Approximately 10%–20% of symptomatic patients with cholecystolithiasis have choledocholithiasis, resulting in infection, abdominal pain, jaundice, and biliary pancreatitis. This study aimed to determine whether a dysfunction in the sphincter of Oddi, which controls the outflow of bile and separates the bile duct from the intestine, leads to a change in biliary microbiota and the occurrence of biliary calculi.

**Methods:**

Forty patients with cholecystolithiasis and choledocholithiasis were prospectively recruited. Bile specimens were obtained, and biliary pressure was measured during and after surgery. The collected specimens were analyzed with 16S rRNA gene to characterize the biliary microbiota. The risk factors of common bile duct calculi were analyzed numerically combined with the pressure in the sphincter of Oddi.

**Results:**

Different biliary microbiota were found in all cases. Patients with sphincter of Oddi dysfunction had significantly increased biliary microbiota as well as significantly higher level of systemic inflammation than patients with normal sphincter of Oddi.

**Conclusions:**

The systemic inflammatory response of patients with sphincter of Oddi dysfunction is more severe, and their microbial community significantly differs from that of patients with normal sphincter of Oddi, which makes biliary tract infection more likely; furthermore, the biliary tract of patients with sphincter of Oddi dysfunction has more gallstone-related bacterial communities.

## 1 Introduction

Gallstone disease, a benign disease, has a significantly higher incidence rate in Asian countries, such as China, Japan, and South Korea, than in western countries, and it is the main cause of cholecystitis, pancreatitis (Everhart and Ruhl, 2009), and other complications and increases the risk of cardiovascular disease (Upala et al., 2017; Shabanzadeh et al., 2017). Approximately 10%–20% of symptomatic patients with cholecystolithiasis have choledocholithiasis (Yan and Zheng, 2017). Abnormal biliary tract structure, high fat diet and poor sanitation conditions are considered to be the causes of common bile duct stones (CBDS) (Tazuma, 2006). Some scholars consider inflammation, infection, and bile duct stones to be closely related (Villalabeitia et al., 2017), but few studies have presented direct evidence. The sphincter of Oddi (SO) controls the unidirectional outflow of bile and separates the bile duct from the intestine, which is populated by bacteria (Zhang et al., 2008).

Sphincter of Oddi dysfunction (SOD) is a benign non-tumoral disorder of the major papilla. It occurs mainly after cholecystectomy but can also occur before surgery. The underlying pathophysiology is not clear, but it is proposed that the gallbladder serves as a reservoir to off-load increased pressure in the common bile duct occurring during sphincter spasm, and its removal leads to the unmasking of preexisting SOD (Lisbona, 1992). Another possible explanation is that there is the alteration of the sphincter of Oddi motility because of the severing of nerve fibers that pass between the gallbladder and the sphincter of Oddi *via* the cystic duct (Luman et al., 1997). However, SOD has also been observed in patients with intact gallbladders, which suggests that some other process may be involved (Choudhry et al., 1993). Biliary pain and biliary colic are the most frequent symptoms. In about half of the cases, there is a fibrotic stricture of the sphincter of Oddi, probably secondary to the passage of biliary stones, while in the remaining half, the syndrome is due to ampullary motility disorders (Boivineau et al., 2022).

Early studies also confirmed that the formation of bile pigment stone in the common bile duct was related to the anatomical abnormality and dysfunction of Oddi sphincter (Wu et al., 2007). In guinea pigs experiment, researches confirmed that a cholesterol gallstone-causing diet may induce SOD, the increasing tension of SO along with its decreasing activity may play an important role in cholesterol gallstone formation. This may be related to the increasing of serum vasoactive intestinal peptide (VIP) and the reduction of cholesterol octapeptide (CCK-8) caused by cholesterol diet (Zhang et al., 2014; Rong et al., 2016). SOD can lead to significant changes in the bile duct microenvironment. Earlier studies have shown that a change in biliary microbiota and inflammatory reaction caused by corrosive intestinal content reflux may be the key to the formation of bile duct stones (Toouli, 2009). Bile duct microbiota and bile components are important components of bile duct microenvironment and play a key role in bile duct stones. Sphincter of Oddi laxity (SOL) is a critical risk factor for the recurrence of choledocholithiasis after surgery. The presence of *Clostridium* may be potentially associated with the recurrence of SOL-induced choledocholithiasis (Zhang et al., 2020).This shows that SOD, bile duct stones and biliary microbiota interact with each other and promote the development of biliary diseases (infection, stones, dysfunction).

Previous studies have identified many potential pathogens associated with biliary tract infection. The colonization of Enterococcus in biliary tract is related to the occurrence and development of primary sclerosing cholangitis (PSC) (Zigmond et al., 2022). Bile homeostasis disorders and microbiota dysbiosis are the key factors of PSC (Huang et al., 2022). The relationship between biliary microbiota and biliary diseases is not limited to inflammation. The biliary microbes showed association with good prognosis in cholangiocarcinoma, but with poor prognosis in pancreatic adenocarcinoma, and vice versa (Kirishima et al., 2022).

At present, little is known about the changes in microenvironment in patients with SOD. Studies have shown that all biliary microbiota in patients with CBDS can be detected in the upper gastrointestinal tract, and the biliary microbiota is more similar to duodenal fluid samples (Ye et al., 2016). Studies have also shown that oral and respiratory microbiota are more consistent in bile samples than intestinal microbiota (Shen et al., 2015). Therefore, in addition to intestinal species, bacteria from the mouth/respiratory tract may also be associated with human biliary tract infection. However, many details of the interaction between microbiota and SOD in bile duct are unclear.

Because SO injury can aggravate the probability of recurrence of postoperative cholangitis and stones, it has attracted more and more attention (Mu et al., 2015). This study aimed to understand the relationship between the systemic inflammatory response in patients with SOD and the difference in microbial flora in the bile duct between patients with SOD and those with normal SO function using 16SrRNA or ribosomal DNA sequence analysis and correlation analysis.

## 2 Materials and methods

### 2.1 Patients

Patients who underwent cholecystectomy, common bile duct exploration, T-tube drainage (laparotomy and laparoscopy) from 2019 to 2021 were selected as participants. The intraoperative bile duct samples were collected prospectively. Bile duct exploration and anterograde measurement of basic SO pressure were both performed using T-tube at the hepatobiliary surgery clinic of Qinghai Provincial People’s Hospital 3 months after operation, and bile duct samples were collected again. This study was approved by the ethics committee of Qinghai Provincial People’s Hospital (ethical approval number: 2018-ZJ-758). All patients signed a surgical consent form. Informed consent was obtained from all individual participants included in the study. This study included patients with emergency diagnosis of CBDS and were confined to our hospital; those with no previous history of endoscopic retrograde cholangiopancreatography or bile duct exploration; those who had no intake of antibiotics or probiotics 3 days before operation (except 30 minutes before operation); those who had no other biliary diseases, such as malignant tumor of the hepatobiliary pancreatic system, biliary malformation, liver cirrhosis, biliary pancreatitis, post-gastrointestinal reconstruction, etc.; patients whose bile samples were collected for the second time and who performed water fasting ≥12 hours before bile duct exploration and did not take antibiotics and probiotics 3 days before bile collection. Patients with long-term use of hormones, antibiotics and probiotics were excluded.

### 2.2 Collection and preservation of specimens

According to the criteria of SOD, the patients were divided into those with SOD (experimental group) and those with normal sphincter of Oddi function (SOF) (control group). A total of 28 specimens were collected. In the experimental group, the intraoperative specimen numbers were SODpre1 to SODpre7 and the postoperative specimen numbers were SODpost1 to SODpost7. In the control group, the intraoperative specimen numbers were SOFpre8 to SOFpre14 and the postoperative specimen numbers SOFpost8 to SOFpost14.

According to the grouping information, the samples were processed as follows: the bile samples were thawed at room temperature before being fully beaten; 1 mL of bile was loaded into a 2-mL sterile anti-adhesion centrifuge tube (Corning, Corning, NY, USA) with a calibrated standard pipette, and the remaining samples were treated as medical waste. The above operation process was carried out on the standard sterile operation platform. The thawed bile waiting for operation was temporarily put into ice and stored at low temperature. The repackaged samples were sent to Wuhan Huada Gene Sequencing Laboratory for examination and finally sent to the test unit for completion by the author himself. They were sent to the laboratory before the residual amount of dry ice reached half and were then transferred to a low-temperature refrigerator (−80°C) for subsequent experimental treatment.

### 2.3 SOD criteria

**Figure 1:**
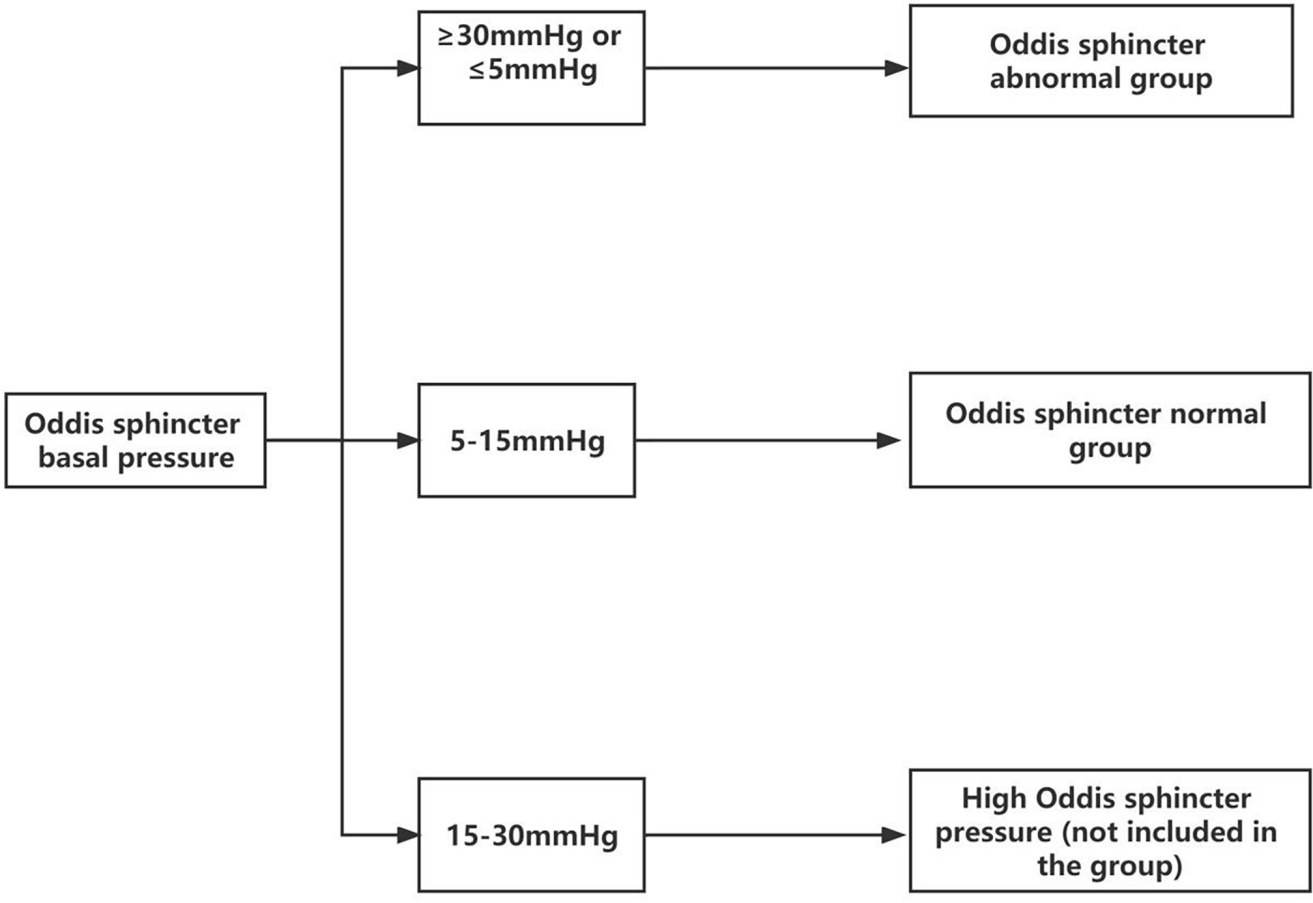
Grouping according to the basal pressure in the sphincter of Oddi.

A common method of sphincter of Oddi manometry (SOM) is to measure the pressure under retrograde cholangiopancreatography. Because of the uniqueness of the experiment itself, the basic pressure of the SO had to be measured by anterograde SOM under the choledochoscope through the T-tube. Owing to the destruction of the integrity of the bile duct, the actual measured SO pressure was too low small to use the reference value of the existing retrograde SOM. The fluid pressure of the T-tube defined for SOD was greater than 30 mmHg. According to this definition, we improved the method of anterograde manometry under choledochoscope through the T-tube. Senior doctors observed the appearance and contractile function of the SO under the choledochoscope (lens body number: mal207; Olympus, Tokyo, Japan) for approximately 10 min to determine whether the SO had poor contraction, sphincter relaxation, sphincter inflammation, fibrosis, dissection, and structural abnormalities. The basal pressure of the SO was measured by anterograde SOM through the T-tube sinus, and the average of three basal pressures was obtained. The basal pressure of the SO was classified as follows: <5 mmHg, lacks motor function; 5–15 mmHg, normal; >15–30 mmHg, slightly increased pressure; >30 mmHg, with excessive motor function or spasm. Those with pressure <5 or >30 mmHg were collectively referred to as the group with abnormal SO (sphincter dysfunction group) (**Figure 1**). Combining the above two methods, the specimens of SOD were included (**Table 1**).

**Table 1:** Samples with sphincter of Oddi dysfunction.

	SOD1	SOD2	SOD3	SOD4	SOD5	SOD6	SOD7
Status	Inflammation	Scar stenosis	relaxation	Fibrosis	Fibrosis	Inflammation with a little fibrosis	Inflammation
Basic pressure (mmH g)	32.8	34.9	4.3	35.0	3.6	36.8	31.5

SOD, sphincter of Oddi dysfunction.

### 2.4 DNA extraction and sequencing

DNA extraction and sequencing were completed using the Huada gene sequencing platform. Microbial DNA extraction reagent was used to extract the genetic material of the bacteria, and then, the DNA was quantitatively analyzed by nanodrop. Finally, the extract was added into agar with different solubilities and electrified to analyze its quality. To control the bias of the polymerase chain reaction (PCR), the obtained DNA samples were divided into three test tubes for PCR low-magnification amplification. Three tubes of PCR amplification products of the same sample were mixed, and 30-ng high-quality DNA samples were obtained, to which corresponding primers were added and the mixture was placed into the PCR amplification container. The parameters of the machine were set, and low-magnification amplification was carried out. Then, the amplified solution was separated and purified with DNA attraction magnetic beads and the previously mixed primers. The purified genetic material was marked and dissolved in elution buffer. The above procedures were completed before sequencing. The purified material can also be subjected to repeat quality control. The reagent was Agilent 2100 (Agilent Technologies, Santa Clara, CA, USA). An appropriate platform, generally HiSeq platform, was selected for the sequencing of qualified samples subjected to repeat quality control.

### 2.5 Processing of sequencing off-line data

#### 2.5.1 Information analysis process

The data generated from the sequencing machine were further filtered, and messy data were sorted out, leaving the group with high-quality bases for further analysis. The overlapping fragments between sequences were spliced into tags; then, the tags were cluster into operational taxonomic units (OTU), which were compared with gene database and annotate species so as to facilitate the subsequent biological information analysis, association analysis, and model prediction.

#### 2.5.2 Data filtering

The off-line raw sequencing data were processed to obtain clean data using the following steps:

Reads that can match the primers were intercepted by the Cutadapt version 2.6 (National Bioinformatics Infrastructure Sweden, Uppsala, Sweden) in order to obtain the fragments of the target region. Using the window average elimination minimum quality method, a window with a length of 30 BP was set to cut off all base-end sequences with a window average quality −20 and directly deleted the base pairs with a final length <75% of the original length. Reads that contained N were removed. Ten consecutive ATCG reads (due to low complexity) were removed to obtain the final clean data.

#### 2.5.3 Tag connection

The procedures for tag connection were the following:

USEARCH OTU clustering method: Tags required OTU clustering directly and DNA sequence splicing with Flash software. Using the overlap between two bases, multiple bases were spliced into tags in a hypervariable region. The splicing conditions must meet the following conditions: the minimum length of matched base sequence should be 15 BP and the error rate of the overlapping regions of the two base sequences must be controlled within ≤0.1 2.

DADA2 OTU clustering method: Tags were processed in DADA2 package and were not used as quality control index.

#### 2.5.4 Statistics of the OTU clustering results

USEARCH: The OTU was generated according to 97% sequence similarity clustering.

DADA2: The amplicon sequence variant (ASV) was generated by clustering the denoised sequence with 100% similarity, which is collectively referred here as the OTU.

USEARCH: USEARCH version 7.0.1090_i86linux32 was used to cluster the spliced tags and generate the OTU. The specific splicing process was as follows: (1) the base sequences with a similarity of more than 97% in the UPARSE program was clustered to obtain the representative sequences of the OTU; (2) by subtracting the chimeras generated after low-cycle PCR amplification from the OTU representative sequence we wanted to obtain; (3) using the USEARCH-GLOBAL program tool, tags were compared with the OUT representative sequence we formed, so as to obtain the statistical table of OTU abundance of each sample.

DADA2: The DADA2 method in the QIIME2 software was used to denoise and obtain the ASV, which is a 100% similar sequence, and then the feature table (collectively referred to as ASV/ASV, etc.) was obtained. The main process is as follows: (1) the filtered double-ended sequence was imported by QIIME tools import; (2) using the QIIME DADA2 denoise paid command, the imported double-ended sequence was constructed based on the DADA2 method; (3) the QIIME tools export was used to convert the feature table into a format that can be viewed directly.

### 2.6 OTU notes

We compared the obtained OTU representative sequences with the existing microbial data with RDP Classifier version 1.9.1. After comparison, the OTU sequence was annotated with the species (the confidence value of species annotation was set to 0.6); the species annotation bases that were not in the database or within the scope of our study were deleted, and the remaining OTUs were used for statistical analysis.

## 3 Results

### 3.1 Comparison of general conditions and relevant laboratory indexes between the two groups

The preoperative laboratory indexes of experimental group (with SOD) and control group (with normal SO) were statistically processed. There were 7 patients in the experimental group (sex, 3 male and 4 female; age, 60–77 years) and 7 patients in the control group (sex, 4 male and 3 female; aged, 35–76 years). Except for age and leukocyte indicators, no other indicators had normal distribution or uneven variance. We used the median and quartile for statistical description and performed the rank sum test. There was a significant difference in leukocytes between the two groups (P ≤ 0.05), indicating that the systemic inflammatory response of the patients in the experimental group might be more severe. There was no significant difference in other indexes after statistical treatment (P≥0.05) (**Table 2**).

**Table 2:** General characteristic of the two groups of patients and the first preoperative related laboratory indicators.

	SOD	SOF	P value
Sex (male/female)	3/4	4/3	**−**
Age (years)	68.29±6.47	56.86±14.59	0.084
Leukocyte (109/L)	12.36±3.31	4.30±1.44	0.006
High-sensitivity C-reactive protein	1.84 (0.40/4.04)	0.26 (0.200/2.99)	0.259
Total bilirubin (μmol/L)	42. 00 (32.30/91.00)	42.40 (25.60/50.50)	0.710
Direct bilirubin (μmol/L)	15.00 (10.10/46.00)	17.40(8.00/22.40)	0.902
Alkaline phosphatase (U/L)	537.00 (120.00/831.00)	196.00 (138.00/430.00)	0.535

Data are presented as n/n, mean, deviation, median (quartile). SOD, sphincter of Oddi dysfunction; SOF, normal sphincter of Oddi function.

### 3.2 Sample sequence and ASV/OTU

**Figure 2:**
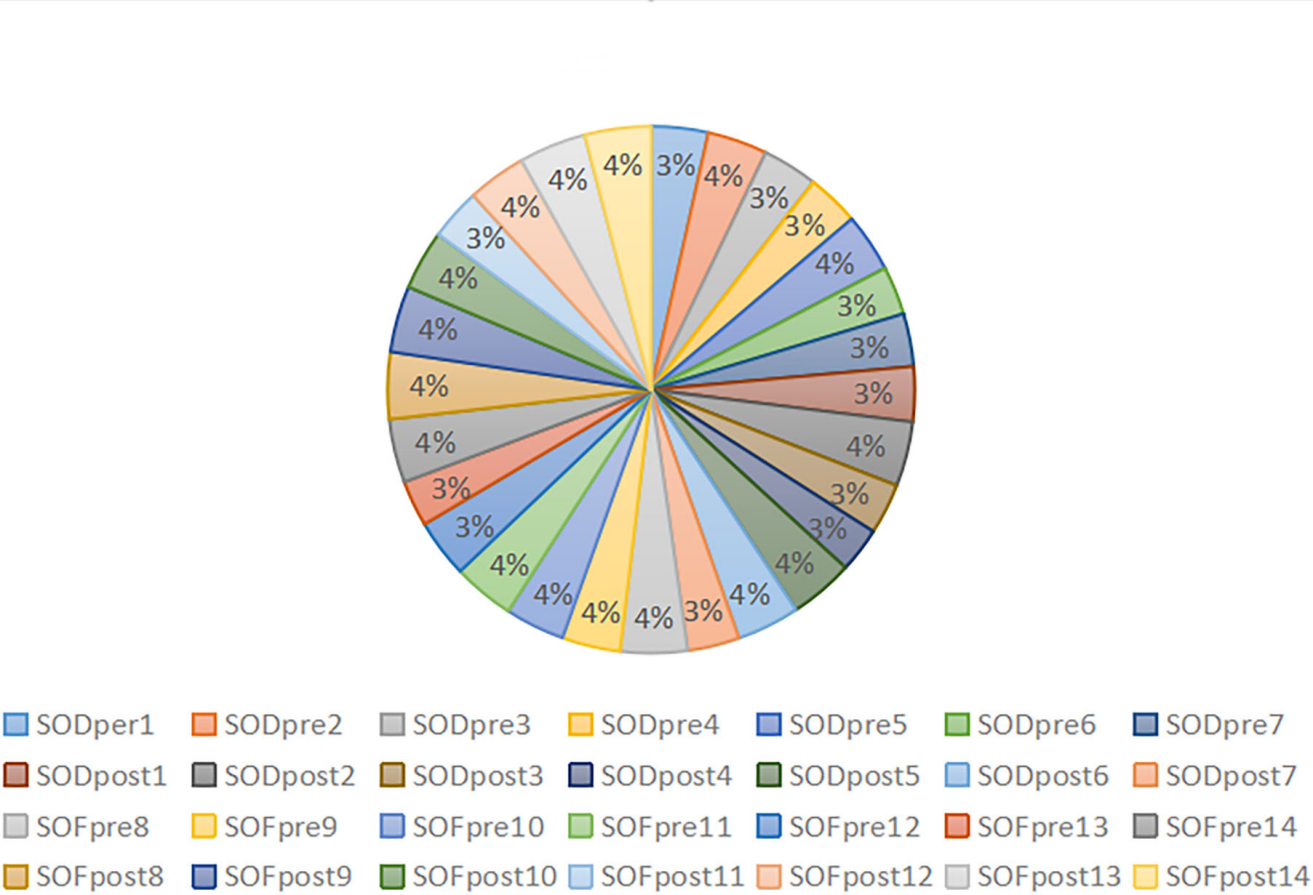
Percentage of valid base sequence entries in each sample.

**Figure 3:**
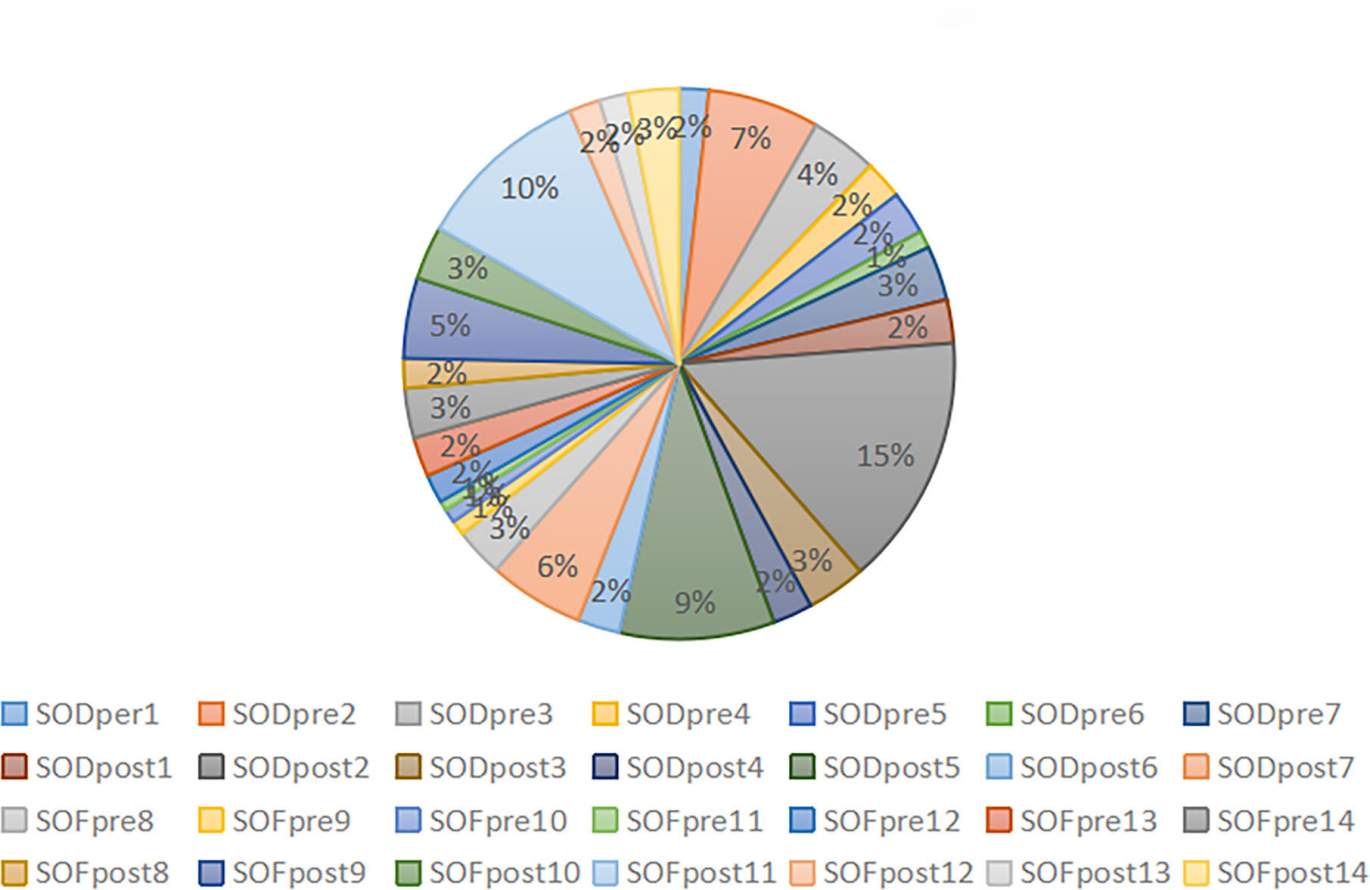
Proportion of amplicon sequence variant/operational taxonomic unit per sample.

After obtaining clear sequencing data, we used the USEARCH method to cluster and merge the base sequences with recognition ≥97% into an OTU. Finally, we obtained the high-quality sequence 1717983. After removing the ASV sequence with a sequence mean of 1, we collected the final effective sequence of 1706982, with an average of 60,963.64 ± 66,98.753. The data output status and ASV/OTU results of each sample are presented in **Table 3**. The percentage of effective base sequence of the samples in the total and the percentage of ASV/OTU number of samples in the total are represented by statistical pie charts (**Figures 2**, **3**).

**Table 3:** Sample data volume output and ASV/OTU result statistics.

Sample name	High-quality sequence	Final valid sequence	Tag average length (PB)/standard deviation	Number of tags	ASV/OT U
SODpre1	59,650	59,517	428/1	59,474	24
SODpre2	62,950	62,786	406/3	62,710	90
SODpre3	58,096	57,792	428/1	57,708	54
SODpre4	55,241	54,951	428/1	54,863	31
SODpre5	62,053	61,492	428/2	61,400	35
SODpre6	51,101	50,765	428/1	50,641	14
SODpre7	55,433	55,250	416/10	55,193	44
SODpost1	58,106	57,551	425/8	57,092	35
SODpost2	68,595	67,783	421/8	60,712	203
SODpost3	53,497	53,314	429/3	53,133	48
SODpost4	47,160	47,060	410/8	46,858	32
SODpost5	67,404	67,167	427/7	62,989	125
SODpost6	66,504	65,826	428/2	63,988	34
SODpost7	54,630	54,443	429/2	54,385	77
SOFpre8	70,581	70,062	428/2	62,093	39
SOFpre9	61,557	61,071	428/2	61,023	12
SOFpre10	62,828	62,481	428/1	62,329	11
SOFpre11	64,931	64,511	428/3	63,544	8
SOFpre12	61,410	61,003	428/1	60,811	23
SOFpre13	48,654	48,286	425/8	48,241	32
SOFpre14	67,606	66,633	428/2	62,529	40
SOFpost8	70,305	69,905	424/7	62,211	24
SOFpost9	69,876	69,534	423/10	63,288	65
SOFpost10	62,788	62,406	423/10	60,812	43
SOFpost11	54,307	54,014	422/9	53,315	142
SOFpost12	61,965	61,672	425/8	61,603	25
SOFpost13	70,869	70,290	427/5	63,869	23
SOFpost14	69,886	69,417	427/6	61,887	41

The effective base sequence in each sample was basically uniform, which indicated that the total amount of bacteria in each sample was relatively uniform. After clustering the effective base, the number of OTU/ASV in each sample was quite different. In the case of ASV/OTU in each sample, SODpre2 (7%), SODpost2 (15%), SODpost5 (9%) and SOFpost11 (10%) had high proportions. The species richness of these samples was high. Whether the increased richness was caused by individual differences or could represent the overall situation of this group of samples should be further analyzed in combination with diversity analysis and species annotation.

### 3.3 OTU statistical analysis

#### 3.3.1 Species accumulation curve of population sample

**Figure 4:**
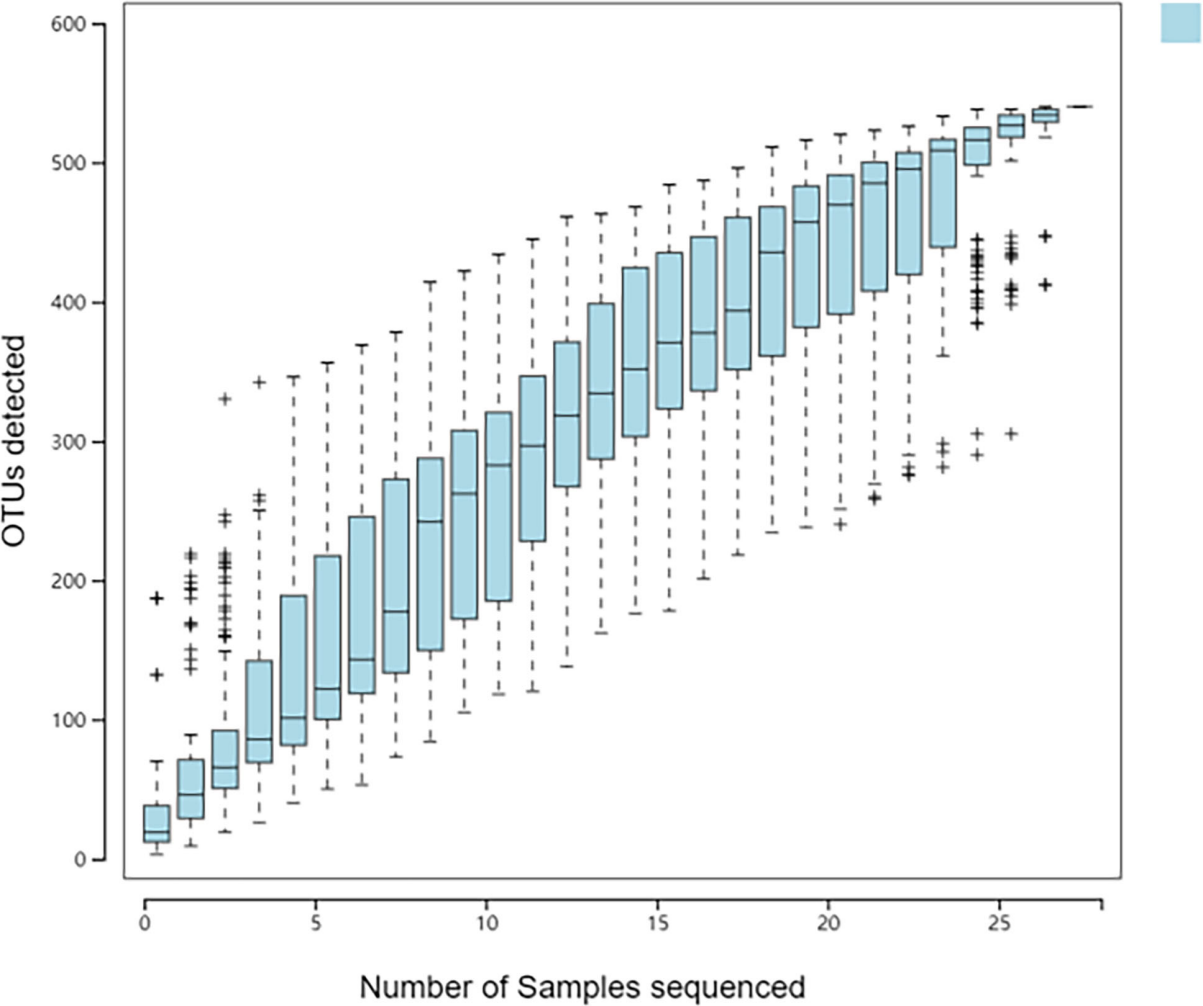
Cumulative species curve of the population sample.

There are many methods to measure and predict species richness in samples, and the species accumulation curve is one of the best ways. Its abscissa is the sample size, and its ordinate is the number of species detected. The blue shadow indicates the confidence interval. The ordinate on the curve increases with the abscissa value. When the ordinate reaches a certain value, the curve tends to be horizontal. Graphically, when the sample size reached 30, the curve was almost horizontal. With further increase in sample size, no new species appeared (**Figure 4**). It showed that the samples in our study had met the requirements of the study, and the number and sequencing depth of the samples could reflect the structure of the microbiota in the samples.

**Figure 5:**
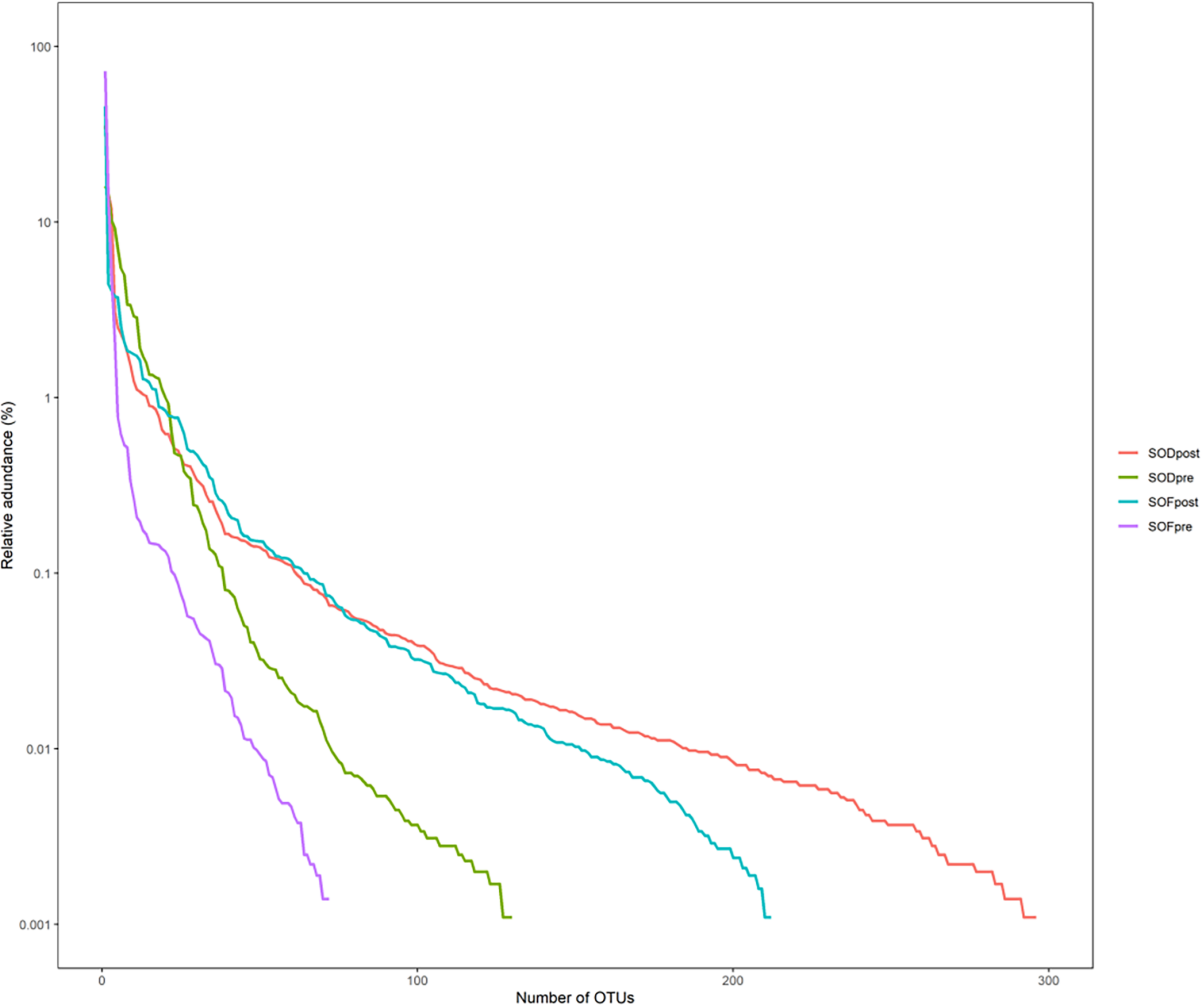
Rank abundance curves of groups.

The rank abundance curve arranges the ASV/OTU in each group in the order of quantity and size and then uses the abundance value of each group as the ordinate to connect the ASV/OTU in each group with a broken line. SODpost had the highest microbiota abundance, followed by SOFpost, SODpre, and SOFpre, indicating that the species of the microbiota in the SODpost group were the most abundant, whereas the species of the microbiota in the SOFpre group were the least abundant (**Figure 5**). Regardless of whether the increase in the number of biliary microbiota in the experimental group was caused by the entry of intestinal microbiota into the biliary tract after SO abnormality, follow-up analysis should be performed to judge whether the increased microbiota was a common microbiota in the intestinal tract.

#### 3.3.2 Species abundance heat map

**Figure 6:**
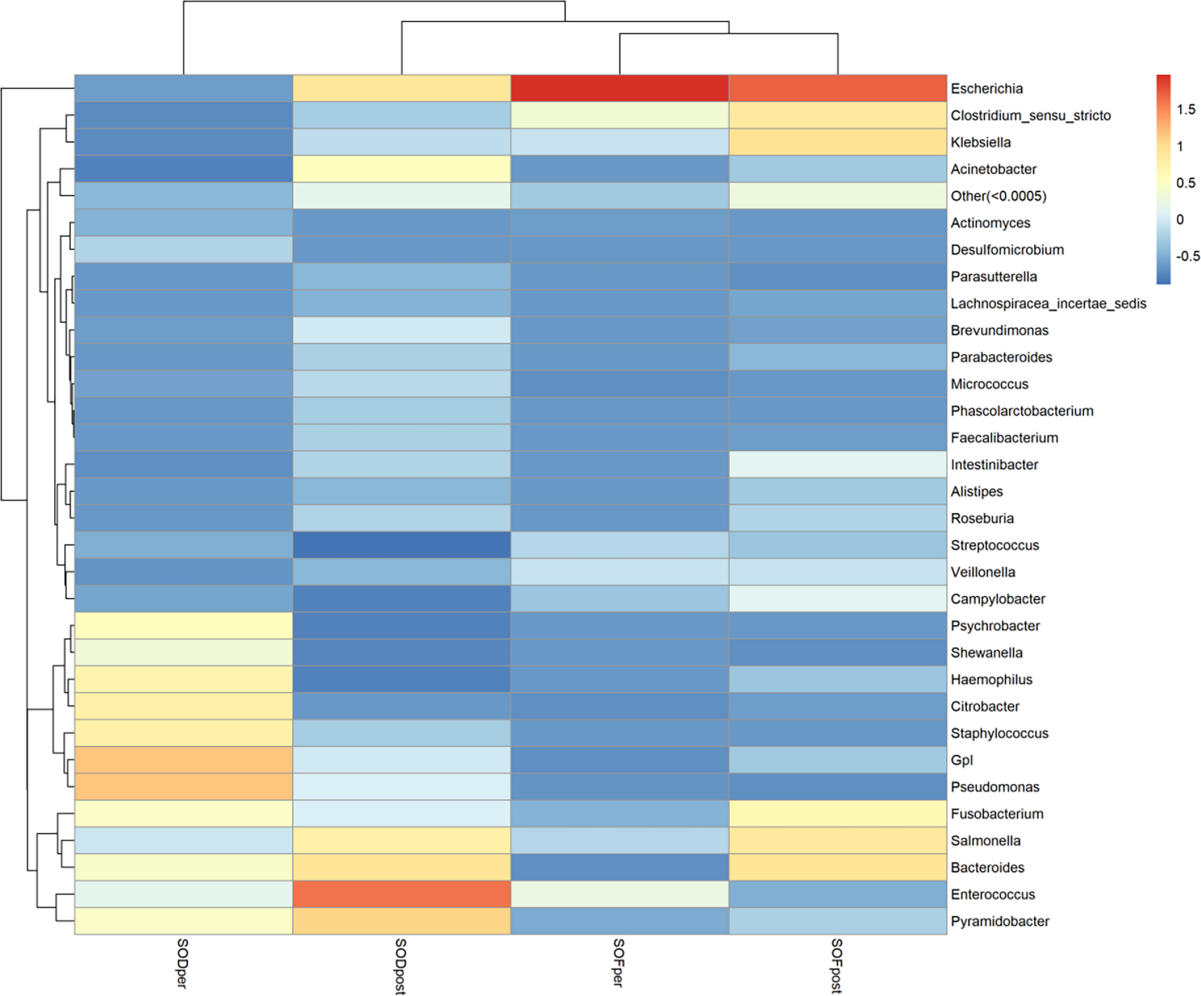
Cluster heat map of species abundance at each genus level.

To find the aggregation and relative abundance of the dominant bacteria in the samples of the experimental group, we annotated the information according to the species at the genus level of all samples. We selected the genus level of the top 32 species in abundance, clustered them from two levels of samples and species, and drew a heat map (**Figure 6**). A redder color indicates more abundant bacteria; blue was a negative value, indicating that the relative content of this bacterium was lower than that of the other groups. As a whole, there were great differences among the microbiota and genera in each group, and the distribution of the dominant microbiota in each group was significantly different from that in other groups. The microbiota of the SODper group was more concentrated in the lower right corner, and the relative abundance value of species was also concentrated in the lower right corner. The sample microbiota in SODpost was relatively scattered at the genus level, and the dominant microbiota was only *Escherichia* (relative coefficient, 1.96), followed by *Clostridium sensu stricto* (relative coefficient, 0.34) and *Enterococcus* (relative coefficient, 0.24). The abundance of microbiota in the samples between SODpost and SOFpost was relatively scattered at the genus level, and there were significant differences in the dominant microbiota between the two groups.

## 4 Discussion

In many Asian countries, including China, the incidence rate of CBDS is much higher than that in some western countries, and the occurrence of CBDS can be affected by the interaction of environmental, genetic, and other factors (Chen et al., 2015). The composition and calcification pattern of gallstones differ, and the main factors involved in stone formation and calcification have not been determined. Current relevant studies consider the supersaturation of bile as the first step in gallstone formation. Therefore, any factor that increases the concentration of cholesterol will increase the possibility of gallstone formation. The relative concentrations of bile salts, lecithin, and cholesterol also determine the physical state of bile (Rudling et al., 2019). In addition to these factors, bacteria also play an important role in the formation of gallstones (Dutt et al., 2003; Gomes et al., 2009; Kose et al., 2018). We previously believed that the bile of people with normal SO may be sterile because the high concentration of bile acids can inhibit bacterial growth. A previous study also found that, compared with patients without SOD, patients with SOD have more types of microbiota (Liang et al., 2016). In the SOD groups, choletropia was common and pathogenic bacteria such as seaweed Schwann bacteria were abundant, while harmless bacteria were decreased. Similarly, some studies on endoscopic treatment of CBDS found that, after the destruction of the SO, the incidence of postoperative cholangitis did not decrease significantly, and the incidence of long-term bile duct stones was as high as 12% to 18.9% (Natsui et al., 2013; Lu et al., 2014). In addition, a recent experiment has shown that intestinal bacteria can translocate to the biliary tract in some cases (Xiao et al., 2021). The abnormal translocation of bacteria changes their settlements. After entering the biliary tract, they grow and reproduce without the restriction of the intestine and then cause related diseases. With the imbalance in biliary microbiota and bacterial translocation, these bacteria cause infection and inflammation under certain conditions. Repeated infection leads to chronic inflammation, which can cause inflammatory cell infiltration, exudation, proliferation, and fibrosis, leading to bile duct stenosis and deformation. This study prospectively studied the microecology of the biliary microbiota in patients with CBDS with normal and abnormal SO. A previous study has shown the role of intestinal microbiota in the pathogenesis of CBDS (Molinero et al., 2019). In this study, we applied high-throughput sequencing technology and bioinformatics analysis methods to extract the genomic information of biliary microbiota and describe the characteristics of microbial community in the biliary tract. The clinical data of this experiment showed that the leukocyte count in patients with SOD are significantly higher than those in patients with normal SO. This is probably related to cholangitis in patients with SOD. We found that patients with abnormal SO in CBDS have more microbiota in bile, which are more likely to cause systemic inflammation. Therefore, the occurrence of CBDS may also be closely related to the abnormal microbiota in the biliary tract. Microbial species in bile also need to be balanced to prevent pathogen infection and pathological inflammation associated with gallstone formation (Wu et al., 2013). Further attention should be paid to the management of patients with SOD during hospitalization and the recurrence of long-term bile duct stones. Whether the biliary tract is sterile under normal conditions remains unknown and thus should be studied further.

SOD can promote the reflux of intestinal contents to the common bile duct. The implantation of bile duct microbiota not only causes local inflammation, but also affects bile metabolism to a great extent (Agha et al., 2017). However, whether there is a real association between SOD, biliary microbiota and choledocholithiasis needs to be further confirmed by a multi sample randomized controlled trial. It is worth noting that the composition of biliary microbiota in the SOD group we studied is generally consistent with the researches of Liang (Liang et al., 2016), Pereira (Pereira et al., 2017) and Zhang (Zhang et al., 2020). In this study, we pay more attention to the diversity of postoperative biliary tract metabolism. We conclude that compared with patients with SOF, the metabolism of patients with SOD is more concentrated in the genera of Escherichia coli, Clostridium and Enterococcus. This indicates that the bile of patients with SOD is infected with more intestinal pathogens than that of patients with SOF, and the increase in the abundance of these metabolism can be used as a biomarker for the recurrence of common bile duct stones. In addition, studies have shown that Clostridium is related to human bile salt metabolism (Ridlon et al., 2006). This may be one of the reasons for the formation of choledocholithiasis caused by metabolism. In conclusion, whether the increase in the abundance of these metabolism causes the recurrence of common bile duct stones still needs further follow-up and larger sample research.

This study has several strengths. It compared the microbiota and inflammatory indexes between groups with normal and abnormal SO in order to explore the changes in bile duct microenvironment in patients with CBD. The results clarified the microbiota structure of

SOD and described the microenvironment differences between patients with SOD and those with normal SO in detail. The changes in bile composition and the increase in inflammatory level both indicate that patients with abnormal SO have more severe symptoms of cholangitis. Controlling infection and close monitoring have a certain clinical value for preventing the recurrence of bile duct stones in patients with SOD. However, this study has some limitations. First, although the bile samples collected in this experiment were qualified samples, collecting bile from healthy people for comparison is impossible because of ethical concern. Second, the use of intraoperative antibiotics will inevitably affect the microbiota in the bile duct, and the impact of diseases other than CBDS on the microbiota in the bile duct cannot be ruled out. In addition, our sample size is small; thus, a multicenter cohort study should be performed in the future to assess the stability of the observed changes in the bile microbiome.

In conclusion, this study proved that SOD significantly changed the abundance of biliary microbiota and may be related to the occurrence of cholangitis. The study of biliary microecology will provide more scientific basis for the diagnosis, treatment, and stone recurrence in patients with CBDS in the future.

## Data availability statement

The date presented in the study are deposited in the DRYAD repository, accession number: doi: 10.5061/dryad.2rbnzs7s5.

## Ethics statement

The studies involving human participants were reviewed and approved by Ethics Committee of Qinghai Provincial People’s Hospital. The patients/participants provided their written informed consent to participate in this study.

## Author contributions

LL was the patient’s doctor and contributed to manuscript drafting. ZZ handled the revision of the manuscript for important intellectual content. XH and JW contributed to manuscript drafting. LL and ZZ contributed equally to this work. All authors agreed to be accountable for the content of the work. All authors contributed to the article and approved the submitted version.
